# Characteristics of being hospitalized as a child with a new diagnosis of type 1 diabetes: a phenomenological study of children’s past and present experiences

**DOI:** 10.1186/s12912-014-0051-9

**Published:** 2015-01-17

**Authors:** Else Mari Ruberg Ekra, Tora Korsvold, Eva Gjengedal

**Affiliations:** Department of Global Public Health and Primary Care, University of Bergen, Kalfarveien 31, Bergen, N-5018 Norway; Department of Health and Nursing Sciences, Faculty of Health and Sport Sciences, University of Agder, Jon Lilletuns vei 9, Grimstad, N-4898 Norway; Queen Maud University College of Early Childhood Education, Thrond Nergaards veg 7, Trondheim, N-7044 Norway

**Keywords:** Childhood, Vulnerability, Agency, Hospital environment, Lifeworld phenomenology, Chronic illness

## Abstract

**Background:**

Our understanding of children and childhood has changed over the last few decades, which may have an impact on children’s conditions in hospitals. Children’s rights have been strengthened by the “Convention on the Rights of the Child” and ward regulations. The aim of this Norwegian study was to identify potential characteristics of children's lived experience of being hospitalized diagnosed with type 1 diabetes today and from a retrospective view in the period 1950–1980, despite the many obvious external changes.

**Methods:**

This study presents a further analysis of data from two previous phenomenological studies. The first had a retrospective perspective, and the second assumed a contemporary perspective. Twelve adults and nine children who had been hospitalized for newly diagnosed type 1 diabetes at the age of approximately 6–12 years old participated. The adults relayed narratives from their childhood memories through interviews, and the study with the children was designed as a combination of observations, in-depth interviews, and photographs. A hermeneutic phenomenological method was used in the analysis.

**Results:**

The analysis revealed a meaning structure that described a *tension between vulnerability and agency* in the experiences of being hospitalized as a child, both past and present. The experiences may further be characterized as *alienation versus recognition* and as *passivity versus activity*, relating to both the hospital environment and the illness.

**Conclusions:**

To a greater extent than ever, children today tend to experience themselves as active and competent individuals who can manage their own illness. Previously, children seemed to experience themselves as more vulnerable and less competent in relationship to their environment and illness. Presently, as before, children appear to desire involvement in their illness; however, at the same time, they prefer to share responsibility with or hand over responsibility to adults. However, living with diabetes was and remains demanding, and it affects children’s lifeworld. Balancing the children’s vulnerability and agency seems to be the best way to care for children in hospitals. In this article, we thus argue for a lifeworld-led approach when caring for hospitalized children, paying attention to both their vulnerability and agency.

## Background

In this Norwegian study, we will contrast children’s lived experiences of being hospitalized with type 1 diabetes today with those of the period 1950–1980. The study is based on a further analysis of data from two previous phenomenological studies, one retrospective [1] and one contemporary [2], which aimed to explore how physical and social environments influence children’s experiences of being ill and hospitalized. Children with type 1 diabetes were used as an illustrative group in these studies. Type 1 diabetes is a common chronic disease that is often acquired during childhood, and the occurrence of diabetes has increased among children [3]. Diabetes treatments, particularly medical equipment, have improved in recent decades [4,5], which may affect patients’ experiences. The main findings of the retrospective study emphasized the experience of a hospital stay as *children in an adult world*. The participants encountered an unfamiliar and challenging place, abandoned in an adult hospital community with a serious and exhausting illness [1]. The contemporary study revealed the children’s hospital stays as representing *a change through recognition and adaptation*. The children perceived the environment as strange, but still comfortable, and they gradually adapted to a new life with a chronic illness [2].

The view of and knowledge about children and childhood have changed over the last few decades. Children’s rights have been strengthened, which may affect their conditions in the hospital.

Previously, hospital visiting hours were restricted; until the 1970s, children were not allowed to have their parents to stay overnight with them [6], and the environment was not necessarily adapted to children [2]. Despite various official recommendations regarding the welfare of children in hospital [7,8], change proceeded slowly [9]. In 1950, there were only three children’s wards in Norway [10]; even during the 1970’s, many children still had to stay in wards that were designed for adults [8]. The first official Norwegian guidelines for hospitalized children were written in 1979 [11].

Traditionally, childhood has been characterized as a period of dependency, immaturity, and incompetence. Children’s position in society has therefore been subordinate and marginal [12]. Developmental psychology and certain subfields of sociology have been future oriented regarding children, focusing on children as human “becomings” (adults) rather than human “beings” [13].

The social status of children has changed over time and is now supported by the “Convention on the Rights of the Child” [14], which includes protection, provision, and participation in matters that affect them, and children’s hospital wards are guided by regulations and standards [15-17]. “The new social study of childhood” is an interdisciplinary research field that considers children as social actors [18]. This approach requires a shift in perception in research, from seeing children mainly as objects to regarding them as subjects whose experiences and knowledge are valuable [12,18,19]. Until recently, children’s voices have been neglected in research, and parents or professionals have spoken on behalf of children [20]. During the last few decades, studies have been performed *with* instead of on children. These studies have provided new and important knowledge about, for instance, children’s experiences with regard to the hospital environment [21-23], communication [24,25], consultation [26], short-term illnesses [27,28], chronic illnesses in general [29], and diabetes [30,31].

However, few studies have focused on the implications of the changed view of children and whether changes in treatment regimens have altered the experiences of being ill and hospitalized as a child. No studies have been found that illuminate commonalities across different time periods. Furthermore, studies that use a phenomenological lifeworld perspective in an attempt to understand changes in a child’s “world” when hospitalized are rare.

The lifeworld is the human world of lived experience; it is a concrete, pre-reflexive world that we take for granted and share with other human beings [32,33]. According to Merleau-Ponty [34], we inhabit the world as bodily subjects, and the world is perceived and understood through the body. Normally, the child has a pre-reflective relationship with the body. The lived body is completely intertwined with his or her existence; consequently, the child tends to compare the world with his or her physical body size or to combine his or her identity with the body [35]. Existential dimensions of the lifeworld, such as embodiment, temporality, spatiality, inter-subjectivity, and mood, are all intertwined aspects of human lived experiences. In particular, mood influences the other dimensions [36]. A lifeworld approach, with its experiential perspective, gives a broader understanding of the meaning and complexity of health and illness than does a biomedical model [37-39]. Taking this as a starting point, Galvin and Todres [36] suggest a lifeworld-led care model considering these existential core dimensions, which provide a holistic perspective and contribute to humanizing clinical practice. Lifeworld-led care requires that health professionals are open and sensitive to their patients’ lifeworld [36].

### Aim

The aim of this study was to identify potential characteristics of children's lived experience with being hospitalized diagnosed with type 1 diabetes today and from a retrospective view in the period 1950–1980 despite the many obvious external changes.

## Methods

The main features of phenomenological research are that it always begins with the lifeworld, requires a first-person’s perspective and assumes experiential material [38,40,41]. The current study is based on and is a further analysis of data from two previous phenomenological studies, which have been presented elsewhere [1,2].

### Participants and context

The participants were recruited from two Norwegian hospitals with adult diabetes outpatient clinics and children’s wards with an outpatient clinic. Diabetes nurse specialists and staff nurses selected a purposeful sample based on the following inclusion criteria; hospitalized as children at the age of 6–12 years (for adults hospitalized between 1950–1980) and a recent diagnosis of diabetes (Table 1). Twelve adults and nine children participated.

**Table 1:** **Data of the participants (all names are fictional)**

**Retrospective study**					**Contemporary study**				
**No**	**Name**	Age*	Date**	**Ward**	**No**	**Name**	Age*	Date**	**Ward**
1	Aud	12	1964	Medical ward	1	Maria	7	2008-10	Children’s ward
2	Marit	10	1980	Children’s ward	2	Therese	12	2008-10	Children’s ward
3	Bjarne	8	1963	Medical ward	3	Johan	12	2008-10	Children’s ward
4	Trond	5	1975	Children’s ward	4	Andre	8	2008-10	Children’s ward
5	Grete	12	1968	Medical ward	5	Henning	12	2008-10	Children’s ward
6	Berit	6	1970	Medical ward.	6	Lars	9	2008-10	Children’s ward
7	Tove	8	1974	Medical ward	7	Emma	11	2008-10	Children’s ward
8	Karin	11	1968	Medical ward	8	Eva	11	2008-10	Children’s ward
9	Hans	7	1971	Medical ward	9	Mads	10	2008-10	Children’s ward
10	Mona	7	1972	Medical ward					
11	Liv	8	1956	Medical ward					
12	Randi	10	1947	Medical ward					

*Age (years).

**Date of hospitalization and initial diagnosis of diabetes.

The adult participants were admitted to six different hospitals; only two of them had stayed in children’s wards. Several of the participants stayed in four- to ten-bed rooms, together with adults, and had little opportunity to play or do schoolwork. Food was served in the patient’s room. Parents usually visited their children daily, but only in one case was the mother permitted to stay overnight with her child.

The children were admitted to children’s wards in two different hospitals. Both children’s wards had facilities for playing, doing schoolwork, and dining. The rooms had one or two beds, a TV, and an attached bathroom. There was child-related décor on the walls and outdoor spaces. The children had one of their parents present all the time and received visits from family and friends.

### Design and data collection

The retrospective data were collected through in-depth interviews in which the adults relayed narratives from their childhood memories. A mixed data collection, designed as a combination of observation, in-depth interviews [40], and photographs taken by the children [42,43], was used to gain insight into the children’s lifeworld in the contemporary study. The children met with the first author in different contexts in the hospital, and each child was interviewed twice with one of their parents present. The pictures taken by the children after the first interview were the basis for the second interview and were valuable in reminding them of situations and rooms they had experienced.

### Analysis

The analysis presented in this study aimed to explore characteristics of the children’s experiences, both past and present. A hermeneutic phenomenological approach was used, meaning that the findings reflect a process of description and interpretation [40].

The text material consists of verbatim transcription of the interviews in both studies and field notes from the contemporary study. These data were analyzed in accordance with van Manen’s [40] description of thematic analysis.

With the new study aim in mind, the main themes of the previous studies were first reflected on. Initially, the differences appeared to be more conspicuous than potential commonalities. Hence, we had to go deeper into the material. The transcribed text from each interview was then reviewed and reread, notes were written in the margin, and phrases and quotes were underlined to look for potential new emerging themes. Furthermore, the preliminary themes from all the interviews were compared, and a new meaning structure, consisting of two main themes, gradually emerged. The analysis process was characterized by a continuous movement back and forth between the whole and the parts, and we strived for openness and sensitivity to the text [40,44].

### Ethical considerations

The previous studies were designed in accordance with the Declaration of Helsinki [45] and were approved by the hospital’s chief physicians, the Regional Committee for Medical Research Ethics in western Norway (No. 066.08), and the Norwegian Social Science Data Services (No. 19087). The adult participants and all the children’s parents, as well as children older than 10 years, gave written informed consent. All the children gave verbal consent and received written information prepared for children. The participants were assured of confidentiality.

## Results

We will now describe characteristics of children’s lived experience as findings from the past and today.

### Tension between vulnerability and agency

An essential meaning structure revealed an experienced *tension between vulnerability and agency* of being hospitalized as a child, both past and present. This tension may further be characterized as *alienation versus recognition* and *passivity versus activity.*

The hospitalized children seemed to experience themselves both as vulnerable and as actors at the same time, and a tension between these conditions always seemed to be present. In some cases, vulnerability came to the forefront and agency to the background, and vice versa. In the retrospective study, vulnerability was more prominent; however, our findings revealed agency as well. The situation in the contemporary study, however, was reversed.

### Alienation versus recognition

Vulnerability was expressed as alienation in both studies, albeit in somewhat different ways. The participants said they were anxious and insecure when they encountered the hospital. The environment was new and strange, and their body appeared different to them.

Present: I was curious and I was scared… I was so sorry and started to cry… I certainly not wanted diabetes… because of all the pricks and blood glucose tests… However, when I was in the hospital, it was good there as well. (Therese)

In the retrospective study, the participants additionally described a strong feeling of being abandoned when their mothers left the hospital after admission.

Past: I remember very well the situation of being hospitalized… I was very curious… knowning that I had to take injections and I was anxious about that… the first evening my mother went home… when I went to bed I felt so little, so afraid and so lonely. (Marit)

Being diagnosed with a chronic, lifelong disease and experiencing strange body symptoms was described as hard for all the participants.

Present: I had felt unwell the last couple of weeks…, felt tired… lost ten kilos… my mouth was dry… the water tasted strange… I cried and wouldn’t go upstairs (to the children’s ward)… I was so afraid of everything… I was quite shortness of breath and had to take a rest at the top of the stairs… I thought I could die from diabetes. (Johan)

Past: I remember I was very, very thin… I could not run across the gym hall… I had to sit down and breathe… always thirsty, hungry and tired… I slept once or twice a day… I was seriously ill. (Marit)

The findings in both time periods underscored the children’s anxiety and concerns as they relate to the alien body, alertness to body signals, and all of the circumstances of daily living with diabetes. They also described limitations in social relations that were due to arduous treatment regimens.

In the retrospective study, the feeling of being abandoned seemed to increase when children were afraid, insecure, or absorbed in their own thoughts about the treatment and illness, without having their parents nearby. The previously mentioned restrictive dietary regimens, hunger, and painful injections were challenging experiences for the participants.

Past: My mom told me… I do not remember myself… I said something about how I wanted to die because I could not stand… to weigh the food and all that… I was hungry mostly throughout the whole childhood… I remember I was tired of the injections. (Tove)

Living with diabetes is still difficult; given the need for intensive insulin treatment, frequent monitoring of blood sugar levels, and dietary concerns.

Present: Sometimes I get so angry because I have diabetes… I just want to throw the equipment on the floor and escape from the pricks… I cannot just get rid of it… I’m just a little sorry if I think about all of that… it does nothing, but it’s something I do not like. (Therese)

In addition, the hospital appeared to be a foreign place for the children in both studies. In the past, the hospital interior was organized or designed for children to a limited extend.

Past: My mother brought me to the hospital but left soon… I cried and wanted to go home with her… The ward was a very long corridor, with dark, wide doors with a hook handle… I was in a room together with six old women. (Mona)

Although, modern children’s wards are adapted to children, the presence of medical equipment or seeing and hearing other sick children may contribute to scary and alienating experiences of the hospital environment.

Present: I felt it was very dramatic in the treatment room… lots of small things (like syringes etc.) and such huge instruments… I was connected to a big pole… then the pump started to beep… go beyond the treatment room was scary, quite often I heard babies screaming and kids screaming… may be like other people heard when I was in there. (Johan)

By maintaining hospital rules and a rigid treatment regimen, health professionals in the past were perceived as strict by children, who became easily confused and insecure, for example when being spoken to. One of the participants, Tove, often enjoyed running in the hospital corridor and said: *“I think about the staff as a little bit strict. Especially when they asked me to stop running… I was scolded on”.* Although health professionals are currently perceived as friendly to children, they are required to perform treatment regimens and painful procedures that may contribute to alienation.

In the retrospective study, Liv described the experience of standing behind the hospital gate waiting for her mother, saying: *“The best part of the hospital stay was that as sure as anything, my mother came every day, that’s what I looked forward to”.* Previous participants longed for their parents, but participants in both studies felt lonely and separated from family and friends; homesickness was common.

However, both studies also uncovered that the participants experienced recognition, which may play a significant role for agency. Initially, the hospital environment was perceived as unfamiliar to contemporary children; however they gradually became comfortable with its “child-friendly” atmosphere, even if they still depended on having their family nearby.

Present: I got the best room… It had a flat-screen TV, and the others also had a DVD player… I had a PC as well… It had two beds… every other night, my mom and dad was there… it had a shower and a toilet, but these were in another room in the room. (Mads)

Today, children seemed to take their parents’ presence for granted, and they felt safe when they were close to their relatives. One participant, Eva, was dependent on her mother’s presence and said: “*I did not really like to sleep and be alone”.*

Previously, certain children felt comfort and support from adult patients, which may have helped them to become more familiar with the environment. Karin described a woman who became very important to her during the hospital stay; “*The most special experience was that I almost felt myself more attached to her than to my mother, for she was not always there. So afterwards, I almost felt a little guilty”.*

Also, among staff members, there were some exceptions to the strict attitude. The adult participants described health providers who tried to provide support by including the children their daily routines, which made them feel useful. The children in the contemporary study also appreciated nurses who played games with them or invited them to help with various tasks.

In summary, both in the previous era and today, the participants felt alienation towards both their own body and their surroundings, and they missed friends, classmates, and family. At the same time, they tried to grasp the recognizable and make the unknown known.

### Passivity versus activity

The participants in both studies described their hospital stay as a break from their daily lives and felt that these experiences of time figured more prominently in their recollections, given the new rhythms of the hospital. The participants in the retrospective study felt that time passed slowly with long waiting periods and boredom due to lack of toys and opportunity to play. They found it difficult to fit into the hospital environment and act according to the hospital rules without their parents. However, children also described different actives they initiated, such as reading books, helping and making contact with fellow-patients, exploring the hospital, taking trips outside etc. Hans enjoyed exploring the corridors, the elevator, and the ambulances and said: *“I could see when the ambulance arrived… when I heard something… I stood at the window… always fun to listen to”*. The participants appreciated window seats so they could keep an eye on the world outside the hospital, and they found a sense of belonging by personalizing their bedside areas.

Past: I made my own little corner… on the bed and top of the nightstand. I used the fold-out tabletop to play with my doll and keep my belongings… The hospital was a little bit strict… Time passed slowly because I had no activity… I felt alone… longing for home. (Berit)

Children today said that they were given various activities in the hospital regarding play and school activities and educational programs. However, several children were told they were in need of rest and retreated to their rooms, especially during the first few days.

Present: It’s not an ordinary school in a way… we may come and go… it’s just fun in the activity room where we create things… I’m on the computer, I read… watch TV… It really has been so lovely here because I have been able to find peace. It is really the best… just to be in my room. (Eva)

In addition, to the creation of more “child-friendly” spaces in the hospitals, medical equipment has been adapted to children. User-friendly technological developments seem to have given children more agency. Instead of receiving insulin from “big” and cumbersome glass syringes, hospitals now use pens or pumps.

Present: I could choose a syringe or pen… I knew the insulin pen from before, and I started right away… but this autumn, I would like to try an insulin pump. (Therese)

However, some of the participants in the retrospective study said they were taught how to inject a needle by first trying it out on an orange or a glove and later on themselves.

Past: What I best remember, I think, is the syringes when I got diabetes. They were so big… those very long needles. They were long and thick…I got a glove filled with cotton wool… tried to prick on it… went around to fellow patients and played nurse (Mona)

Previous, participants described experiences of being passive bystanders to what happened to them. They described a lack of information and understanding; in several cases, the children were excluded from conversations and/or the conversations were too difficult to understand.

Past: I felt so alone, especially when I got hypoglycemia. I got scared because I did not really know what happened. (Aud)

Previous participants became passive when they did not receive enough information, while contemporary children could increasingly address the situation because they knew what to do.

Present: I felt very, very shaky… so I just asked if I could go and get measured… Then, I drank a soft drink and ate two slices of bread, so I administered one unit (of insulin) into my stomach. (Eva)

Today, children are included in educational programs, especially in terms of practical training. In the contemporary study, children were largely regarded as active participants, and their competence seems to have been acknowledged. A book recommended by health professionals, “How to become an expert on your own diabetes” [46], aimed at children and youth, is an example. However, a well-adapted educational program with visual tools seems to be crucial for engaging children. Emma participated in most of the learning program; she said *“I thought it was a little bit boring… it took so long… Sometimes I went to the activity room while they (parents) were at the meetings”.* Emma felt that illustrations, figures, and films were more interesting and simpler to understand.

The contemporary study showed that the children wanted to be involved in their educational programs and treatment. However, they also preferred that their parents received information on their behalf and occasionally took responsibility, for example measuring blood sugar late in the evening or at night, giving injections, or reminding them about injections. When children felt unsure about the treatment regimen during the day, they noted that mobile phone were useful in making decisions together with their parents.

In summary, children experienced passivity and boredom both in the past and present, as well as a need for rest. At the same time, they were curious and eager to learn new things.

## Discussion

This study provides new insight regarding significant features in children’s experiences of being hospitalized by contrasting contemporary with previous experiences. We argue that using a phenomenological lifeworld perspective as a point of departure both methodologically and theoretically gives a deeper and holistic understanding of the experiences.

The findings revealed that both study groups of hospitalized children experienced tension between vulnerability and agency, despite the many external changes that have taken place. Vulnerability may be reflected in the children’s experience of alienation and passivity. A new and somewhat scary environment, as well as strange bodily changes, seem to contribute to these experiences, which also may reinforce each other. Skepticism regarding unfamiliar surroundings and the need for rest due to unpleasant symptoms may lead to reduced activity. Agency, on the other hand, is expressed through recognition and activity. Normally, children are curious and creative and seem to search for the familiar in the unfamiliar [35]. In the study, this phenomenon is demonstrated by the children’s preferences for keeping an eye on the world outside the hospital by looking out of the window, visiting the activity room when the training became too boring, or “building their own corner” in their bed. Hospitalized children seem to live with this tension, for better or worse.

Children’s agency is influenced by their dependency on adults, their unequal power relationship, and adults’ views of children and childhood [12]. Children are also often associated with vulnerability due to their care needs [47] and lack of experiences in different contexts [48]. Therefore, health professionals’ attitudes, views, and knowledge of children may be crucial to how children experience the stay in the hospital and their care.

There has been a shift from perceiving children as immature, incompetent, dependent, and unable to receive information to seeing children as more competent and active participants. While the participants in the retrospective study did not experience having a voice in their treatment regime, the children in the contemporary study were included in practical training and educational programs from the outset. However, it was difficult to engage children in discussions without visual tools. This observation is in accordance with a study by Roper et al. [49], in which it was found that children want to know more about diabetes care, management, and consequences and that the information should be given in an appropriate form in a child-centered context where they can easily ask questions. A study by Højlund [50] found that the type of diagnosis was very important in communication and the social relation between health care professionals and a child patient. Children with a chronic illness were more included in conversations as equal partners, received information, and could be characterized as competent. In other cases, children with more serious diagnoses were seen as vulnerable, and conversations with health professionals were mainly through the parents. This shows that the experience of vulnerability and agency depends on how the children are being met by health professionals.

However, several studies found that children were not always allowed to participate in decision-making [26,51,52]. Although children and their parents were involved in the decision-making processes, children made few decisions on their own [53]. Despite significant changes in children’s social status, their experiences are not always requested. Bjerke [54], in a study of children’s view of agency in child-adult relation within home and school contexts, argued *“that children not necessarily ask for increased independence from adults, but rather to be recognized as ‘differently equal’ partners in shared decision-making processes, where children are being treated with dignity and respect as valuable persons”* (p.93).

Söderbäck et al. [55] calls for a child-centered care approach that involves health professionals being sensitive to the individual child’s voice and experiences and to treat children with dignity and respect in health care settings.

However, giving children a voice is not the same as giving them responsibility for their own situation. In the contemporary study, children appreciated handing over responsibility to their parents at times and sharing decision-making with them. This finding is in accordance with previous studies by Lambert et al. [24,25], who found that children want to waver between being a “passive bystander” and an “active participant” in the communication process, depending on the situation. Children often want to be involved and to participate in and to contribute to shared decision-making. At other times, they prefer a passive and protected role. Coyne and Harder [56] argue for using a situational approach in balancing the protection of shared decision-making with children. Adults may have a tendency to take a protective stand based on the general view of children as incompetent. Health professionals have to see children from an individual perspective and consider children’s desires, rights, and needs.

Today, health professionals consider hospitalized children’s experiences to a greater extent. However, a focus on the disease and its treatment, technology, frequent test and interventions, and education may obscure the attention given to children’s lived experiences. From this perspective, a child’s competence may be overemphasized and his or her vulnerability overshadowed.

An existential view of human beings includes both human agency or freedom and human vulnerability and dependency. Having the opportunity to make choices and possess a sense of agency and freedom are closely connected to human dignity [36]. Carel [57], with reference to Heidegger, uses the term “being able to be”, which is related to being human agents in the world, acting, being taken seriously by others, and having autonomy and agency. In illness, our ability to act may be reduced. At the same time, vulnerability may also be recognized as something positive and a strength, depending on whether one initially has received help to face illness and adapt to a situation [57,58].

Children’s vulnerability may be seen as being in contrast to the view of children as competent, active participants. However, being vulnerable is a human condition, and having a body makes us fragile and exposed to risks and harm. There is always a tension between human agency and human vulnerability [36]. Hence, it may be important not to ignore any of these concerns.

Although views and knowledge of children and illness have changed among health professionals, traditional dualistic thinking and the biomedical model are still embedded, and the need to consider the lived body may be obscured. Findings from our previous studies [1,2] underscore the mutual connection between the body and its environment. Perhaps efforts to improve feelings of recognition are taken more seriously with respect to environment rather than the lived body. However, facing and living with a chronic illness remains demanding, as was underscored by the participants in our studies; this findings is also in line with several other studies [30,59,60].

Galvin and Todres [36] argue for using a “lifeworld-led” care model to broaden the “patient-centered” view of illness and health to ensure the humanization of care. In this approach, health care providers must consider their patients’ experiences as fundamental to caring. To ensure that children feel that their needs are being met in health care settings, there should be a balance between their experiences of vulnerability and agency. Perhaps the development of children’s agency, while it has the positive aspects of focusing on children’s preferences, choices, or decision-making, does not completely consider the lifeworld of children. Our studies showed that being hospitalized and facing a chronic illness affected and changed children’s entire lifeworld [1] and that children’s experiences of illness and treatment influenced their perceptions of their environment [2]. When the body changes, so does the lifeworld [33]. Accordingly, it may be crucial for health professionals to consider this tension and find a balance between hospitalized children’s vulnerability and agency.

### Methodological considerations

Different methods were used for data collection in the retrospective and contemporary studies on which this study is based. However, it was important to gain insight into the participants’ lived experiences, which may be grasped via different methods. Furthermore, there is a difference between a child’s perspective and an adult’s memory of childhood. Adult memories will always be understood and interpreted by and through current perspectives [61,62]. In the retrospective study, we heard voices of critical and reflective adults. Childhood reminiscences may present some challenges. However, the childhood experiences of the participants became turning points in their lives. Episodic memories or individual events that created strong impressions seem to be an important issue [63,64]. The mixed data collection in the contemporary study may be seen as a strength. Being in the context was helpful to become more familiar with the children before the interviews and provided an opportunity to observe them in different situations. The pictures taken by the children were valuable in contextualizing and reminding them of rooms and situations. Children between six and twelve years were chosen, as this is the period after preschoolers but before adolescence, which both may present other challenges. Finally, it may be important to underscore that the study was undertaken in a Western context, which certainly is a limitation. Although the tension between vulnerability and agency is basic human experiences, the content of such experiences will vary both within a culture and in different cultural contexts.

## Conclusion

Despite the occurrence of major changes for children who are hospitalized and newly diagnosed type 1 diabetes, the present study revealed a tension between experienced vulnerability and agency. The study underscored the importance of listening to the children’s voices and taking into account their lived experience. In this article, we have argued for a lifeworld-led approach that considers a balance between children’s vulnerability and agency when caring for hospitalized children, which seems to be the best way to meet the need of hospitalized children. By focusing on the children’s perspectives and by recognizing children as participating actors, it was possible to gain insight into how these children influenced and created their lifeworld in the hospital context, both past and present. As the previous studies were not designed to investigate vulnerability and agency, it may be important to investigate these issues more deeply in a study designed to accomplish such an aim. Further research on how the adults’ childhood experiences have influenced their later lives and encounters with the health care system would also be interesting. Additionally, an investigation of health professionals’ past and present experiences of working with hospitalized children would also be an interesting topic to explore.
